# Effects of traffic noise exposure on corticosterone, glutathione and tonic immobility in chicks of a precocial bird

**DOI:** 10.1093/conphys/coz061

**Published:** 2019-08-28

**Authors:** Romina Flores, Mario Penna, John C Wingfield, Elfego Cuevas, Rodrigo A Vásquez, Verónica Quirici

**Affiliations:** 1 Escuela de Medicina Veterinaria, Universidad Santo Tomás, Av. Ejército Libertador, Santiago, Chile; 2 Programa de Fisiología y Biofísica, ICBM, Facultad de Medicina, Universidad de Chile, Av. Independencia, Santiago, Chile; 3 Department of Neurobiology, Physiology and Behavior, University of California, One Shields Avenue, Davis, CA, USA; 4 Doctorado en Medicina de la Conservación, Facultad de Ciencias de la Vida, Universidad Andrés Bello, Av. República, Santiago, Chile; 5 Instituto de Ecología y Biodiversidad and Departamento de Ciencias Ecológicas, Facultad de Ciencias, Universidad de Chile, Las Palmeras, Santiago, Chile; 6 Departamento de Ecología y Biodiversidad, Facultad de Ciencias de la Vida, Universidad Andrés Bello, Av. República, Santiago, Chile; 7 Centro de Investigación para la Sustentabilidad, Facultad de Ciencias de la Vida, Universidad Andrés Bello, Av. República, Santiago, Chile

**Keywords:** Development, playback, quail, urban ecology

## Abstract

Repeated exposure to traffic noise may be perceived as a succession of stressors, and therefore, noisy urban environments could lead to a state of chronic stress. In developing animals, glucocorticoids can have organizational effects on the hypothalamic–pituitary–adrenal axis in addition to the classic activation effects, so evaluating the effect of traffic noise during development is urgently needed. To our knowledge, to date six studies have investigated the effects of traffic noise on baseline corticosterone (CORT) and/or the stress response in birds during development; however, these studies were performed in nestling (altricial species), where confounding factors (e.g. communication between nestlings and parents) could mask the real impact of traffic noise on stress. In this study, we evaluated the effect of traffic noise (traffic noise group *vs*. rural noise group) on baseline levels of CORT and stress responses in chicks of a precocial bird species, the Japanese quail (*Coturnix japonica*). Because CORT can also decrease glutathione (GSH) levels (antioxidant and neurotransmitter/modulator), secondly by means of path analysis we investigated whether the strength of the association between CORT levels, GSH levels and tonic immobility (TI) varied in relation to treatment. We observed (i) similar baseline levels of CORT in both groups, (ii) a trend toward higher stress response in the traffic noise group (*P* = 0.08), (iii) similar TI duration in both groups, (iv) higher GSH levels in the traffic noise group and (v) differences in the strength and sign of the associations in relation to the treatment (traffic *vs*. rural). We conclude that the acoustic environment perceived during development has implications for physiology and behaviour; as more research is done on this topic, the need for sustainable urban planning will become clearer.

## Teaser

Traffic noise affects behavioural and physiological aspects. These effects may be stronger when the individual is growing. We perform traffic noise play-back experiments in chicks of a precocial bird. We observe differences in corticosterone (stress) and glutathione (antioxidant) levels.

## Introduction

As a result of population growth, urbanization and globalization of transportation networks, noise generated by human activities has increased dramatically over recent decades (Shannon *et al*., 2016). Chronic noise increases certain stress-related pathologies in humans, such as sleep disorders (Fyhri and Aasvang, 2010), cardiovascular diseases (Babisch *et al*., 2006), hypertension (Dzhambov and Dimitrova, 2018) and cognitive and learning disorders (Szalma and Hancock, 2011). In wild animals, urban noise can alter acoustic communication, may preclude individuals from reproducing properly (Warren *et al*., 2006; Halfwerk *et al*., 2011; Schroeder *et al.*, 2012; Potvin and MacDougall-Shackleton, 2015) and can impede perception of crucial auditory stimuli such as predatory threats (Barber *et al.*, 2010; Meillère *et al.*, 2015). In addition to all these auditory constraints, repeated exposure to urban noise may also be perceived as a succession of stressors (Tennessen *et al.*, 2014) and therefore the noisy urban environment could lead to a state of chronic stress (or allostatic overload) with important fitness costs (McEwen and Wingfield, 2003; Bonier, 2012). Thus, clarifying the potential impacts of chronic noise exposure in wildlife is timely and needed, given the nearly ubiquitous presence of anthropogenic noise worldwide and the forecasted global rise in noise-producing infrastructure (Barber *et al.*, 2010).

Responses to stressors typically involve the activation of the hypothalamic–pituitary–adrenal cortex (HPA) axis that culminates in the release of glucocorticoids (GCs) (e.g. Sapolsky *et al.*, 2000; Wingfield, 2003; Romero *et al.*, 2009). The HPA axis is often divided into two components, baseline GC levels and stress response GC levels (Romero, 2004). The former is an approximation of the seasonal baseline level that the animal should maintain to be able to cope with the predictable demands of the current life-history stage (Landys *et al.*, 2006; Bókony *et al.*, 2009), so it reflects long-term adaptation. The latter (stress response: the increase in baseline GC levels to those reached after 30 min) best reflects short-term plastic responses to environmental perturbations (Romero, 2002; Wingfield, 2013). Elevation of GCs triggers emergency responses such as changes in locomotor activity (Breuner *et al.*, 1998), decreased nocturnal oxygen consumption (Astheimer *et al.*, 1992), lipogenesis (Jenni *et al.*, 2000; Piersma *et al.*, 2000), increased foraging (Bray, 1993; Koch *et al*., 2004) and mobilization of body energy resources (Wingfield *et al.*, 1995; Jenni *et al.*, 2000). These responses redirect animals to a life-saving state (‘emergency life-history stage’ Wingfield *et al.*, 1998), allowing them to overcome the source of stress and recover homeostasis in the best possible physical condition.

GCs can have similar effects on short-term behaviour and physiology in developing animals (i.e. activation effects), and there is a growing body of literature across taxonomic groups which suggests that GCs have organizational effects on developing animals (a process known as developmental programming: Mcmillen and Robinson, 2005). For example, animals exposed to elevated levels of GCs during development can experience sustained morphological, physiological, neurological and behavioural consequences (Butler *et al.*, 2010; Matthews, 2005; Nesan and Vijayan, 2005). Developmental stress generally causes sustained elevation of HPA function, so that animals exposed to stress during development respond more strongly to stressors as adults (e.g. Pravosudov and Kitaysky, 2006; Spencer *et al.* 2009; Marasco *et al.*, 2012), but see Love and Williams, 2008). Thus, exposure to stress can be detrimental to nestlings during development, but can also have lifelong and transgenerational effects on reproductive success and survival (Crino *et al.*, 2013).

To the best of our knowledge, only six studies have investigated the effects of traffic noise on baseline CORT and/or the stress response in birds during development (Crino *et al.*, 2011; Crino *et al.*, 2013; Angelier *et al.*, 2016; Casasole *et al.*, 2017; Kleist *et al.*, 2018, Injaian *et al.*, 2019). All of these studies were conducted in the field; three of them (Crino *et al.*, 2013; Angelier *et al.*, 2016; Injaian *et al.*, 2019) conducted traffic noise playback experiments. These studies observed increased basal CORT and stress response (Crino *et al.*, 2011), no change in baseline CORT and decrease in stress response (Crino *et al.*, 2013), no change in baseline CORT and increase of stress response (Injaian *et al.*, 2019), decreased basal CORT and increased stress response (Kleist *et al.*, 2018) and no effect on CORT levels (Angelier *et al.*, 2016; Casasole *et al*., 2017). Although these studies have been pioneers in trying to elucidating the effect of noise on stress, they were performed in altricial species (i.e. nestlings), where parental care behaviour such as provisioning or nest attendance (e.g. Morgan *et al.*, 2010; Crino *et al.*, 2011) and communication between nestlings and parents (Leonard *et al.*, 2015; Lucass *et al.*, 2016) could be affected by the background environmental noise. The causes of such noise (e.g. roads, human presence) and the consequences of human interference (e.g. habitat fragmentation, chemical pollution, light pollution) (reviewed in Fahrig and Rytwinski, 2009) could potentially influence nestling CORT levels (i.e. confounding factors).

In this study, we evaluated experimentally the effect of traffic noise on baseline levels of CORT and stress response in chicks of a precocial bird species, the Japanese quail (*Coturnix japonica*). Because the aforementioned six studies have reported decrease, increase and no effect of traffic noise on baseline CORT levels and stress response, we did not make specific predictions about the levels of CORT we expected to find. We used quail as a species model because in addition to being precocial, this species has been widely studied, and it has been demonstrated that the stressful situations to which they have been subjected during development have effects on the levels of CORT and in their behaviour (e.g. Hayward and Wingfield, 2004; Hayward *et al.*, 2006; Hazard *et al.*, 2008; Calandreau *et al.*, 2011; Marasco *et al.*, 2012).

In addition to the activation effects of GCs mentioned above, CORT can also decrease glutathione (GSH) levels (Patel *et al.*, 2002). GSH is the major cellular redox regulator and antioxidant, protecting cells from damage induced by reactive oxygen species (Lavoie *et al.*, 2017), and is a neurotransmitter/modulator that binds to the N-methyl-D-aspartate receptor (Chin *et al.,* 2006) involved in anxiety, fear, learning and memory (Janáky *et al.*, 1999; Hovatta *et al.*, 2005; Davis, 2011). For example, low levels of GSH have been related to psychosis in humans (Lavoie *et al.*, 2017) and anxiety behaviour in mice (Hovatta *et al.*, 2005); in domestic fowl (*Gallus gallus*), chicks with increased levels of GSH decreased distress vocalizations and increased sleep (i.e. decrease in anxiety behaviour) (Yamane *et al.*, 2007). Recently, Isaksson (2015) proposed that because of the dual function of GSH (antioxidant and as neurotransmitter/modulator), there could be a link between urban environment and anxiety behaviour. So, by means of path analysis we investigated whether the strength of the associations (path correlations) between CORT levels, GSH levels and tonic immobility (TI) varied in relation to treatment (traffic noise *vs*. rural noise). We tested the path that CORT influences GSH (we expected a negative association) and that GSH influences TI (we expected a negative association); finally, we tested the path that CORT influences TI (we expected a positive association). TI is a standard test to measure fear in birds (*sensu*Jones, 1986; Jones *et al.*, 1991). TI has been described as an unlearnt catatonic state, which is thought to be the final stage in a chain of anti-predator behaviour patterns (Jones, 1986; Mills and Faure, 1991). We chose this behavioural test because (i) in a playback experiment in chickens, Campo *et al*. (2005) observed longer TI in the group of hens treated with traffic noise and (ii) several behavioural tests conducted in quail demonstrated that the duration of TI is a reliable indicator of underlying fearfulness (Hazard *et al.*, 2008). Our study is the first to test how traffic noise affects the relationships between CORT, GSH and fear behaviour.

## Materials and methods

### General procedure

Two-week-old quail were obtained from a licensed hatchery (David Vattuone Calle) and were taken to the Colina campus of Veterinary School of the Universidad Andrés Bello, located in a rural area on the outskirts of the city of Santiago, Chile. The quail were weighed, fitted with a coloured plastic ring for individual identification and then assigned to two treatments: (i) ‘Traffic noise group’ (*n* = 20) and (ii) ‘Rural noise group’ (*n* = 20). Pre-treatment body weight did not differ between the rural group (31.83 ± 4.89 g) and the city group (29.43 ± 5.28 g) (*F*_2, 32_ = 1.41, *P* = 0.26). The birds of the two treatments were housed in separate rooms (see ‘Stress procedure’ section) and acclimatized for a period of 2 days. Because quail begin to thermo-regulate at 4 weeks of age (Vassallo *et al*. 2014), the 2-week old quail were placed in groups of three individuals per cage (30 cm × 30 cm × 25 cm). At the end of the experimental period, we measured TI (Day 9) and after that the quail were separated and placed in individual cages. From Day 10 to Day 13, we took blood samples for baseline CORT, stress response and GSH determination.

The floor of the cage was covered with corrugated paper, and the cages were made of mesh so that there was visual and auditory contact between the quail. Food and water were delivered *ad libitum* (e.g. Calandreau *et al.*, 2011). The maximum and minimum room temperatures were monitored with a maximum and minimum thermometer. The maximum temperature of the city noise room (29.87 ± 2.35°C mean ± SD, *n* = 8) was similar to the maximum temperature of the rural noise room (31.00 ± 1.19°C) (*P* = 0.12). The minimum temperature of the city noise room (22.06 ± 1.20°C) was similar to the minimum temperature of the rural noise room (22.97 ± 2.43°C) (*P* = 0.57). The average temperature of the city noise room (25.96 ± 5.52°C) was similar to the average temperature of the rural noise room (27.06 ± 5.57) (*P* = 0.13°C). Quail were under a 12:12 h light:dark schedule (light on at 0800 h). The birds were not handled during the period of noise exposure and only had visual contact with the staff at the time of feeding, which was done in a consistent manner to avoid disturbing the chicks.

### Noise recordings

Recordings of urban noise were carried out in Santiago downtown in avenues (*n* = 5), highways (*n* = 4) and a subway station (*n* = 1) during daytime on 23 October 2017, using a sound level meter (Brüel & Kjær 2250) fitted with an extended frequency range microphone (Brüel & Kjær 4189), a 3-m extension cable and a windscreen. The microphone was placed at a minimum distance of 5 m from the curb in the avenues, about 6 m above the pavement of the highways and at about 4 m from the railway in the subway station. The recordings lasted 5–8 min, and the maximum sound pressure levels attained during each recording were read directly from the instrument and confirmed by calculating the values from the relationship of the amplitude of the noises recorded and a 93.8-dB SPL RMS 1-kHz calibration tone produced by a sound calibrator (Brüel & Kjær 4231) recorded with the sound level meter microphone positioned in the opening of this device. The maximum amplitudes of the urban noises recorded averaged 89.5 dB SPL RMS (range; 88.1–100.8 dB SPL RMS).

Segments of 2-s duration including the intervals of maximum amplitudes of each noise were selected and pasted successively three times to obtain 10-min tracks of each noise. Attention was paid to avoid waveform discontinuities at the pasting points. Linear fade-in and fade-out ramps of 5 s were applied at the onset and offset of each noise, respectively. The 10-min noise tracks were normalized at the same maximum amplitude and presented in random sequence, leaving 3-min silent intervals between successive noise presentations. Traffic noises were played back with a computer with an amplifier (NAD 3029i) and broadcast via a wide range loudspeaker (Dynaudio BM6) during 8 h for 8 days.

### Stress procedure

In order to be able to compare our results with field work that compared CORT levels in a rural site and a site with higher traffic noise, either by playback experiments (Crino *et al.*, 2013, Angelier *et al*., 2016; Injaian *et al.*, 2019) or by being closer to the road (Crino *et al*., 2011; Casasole *et al*., 2017; Kleist *et al*. 2018), we followed the experimental design used by Campo *et al*. (2005), in the sense that we carried out a playback experiment of traffic noise in a rural site.

For a period of 8 days (Day 1 to Day 8) (following Calandreau *et al*., 2011) and during 8 h (from 10:00 am to 06: 00 pm), we played recordings of downtown Santiago to the traffic noise group. The room was acoustically isolated and lined with acoustic foam to avoid reverberation inside the room. The quail of the rural noise group were in an adjacent room with similar conditions, but in this one the animals were not exposed to traffic noise playback. In this room, the background noise from the rural area was audible throughout day and night. The traffic noise group had higher dB (70.05 ± 5.11, range 61.9 to 73.4) than the rural noise group dB (63.33 ± 6.54, range 56.1 to 70.3) (Mann–Whitney *U* test *U* = 9, *P* = 0.01, *N*_traffic_ = 12, *N*_rural_ = 8).

### Body weight, TI duration and blood sampling

Following Calandreau and collaborators (2011), the day after the treatment ended (Day 9), all quail were weighed and tested for TI. For the measurement of the duration of TI, each quail was taken to another room without quail and then placed face up in the hand of one of the experimenters (V.Q.) for 10 s. The experimenter remained silent and practically motionless in the room. A second experimenter (R.F.) stood away from the quail and recorded the time it took for the quail to try to sit up (TI) after 10 s. The experimenter remained silent and practically motionless in the room. If TI was not achieved after five successive attempts, a score of 0 s was obtained. Conversely, if the bird did not recover from TI after 5 min, the test was terminated and a maximum score of 300 s was given.

The procedure for collecting blood samples in quail has been previously described (Calandreau *et al.*, 2011). Each animal was carried individually from the homecage to a novel room containing no other bird. Samples were taken alternating between the traffic noise and rural noise groups. Blood samples (ca. 5–60 μL) were obtained by puncturing the brachial vein with a sterile needle and collecting blood into heparinized micro-hematocrit capillary tubes. Following Calandreau and collaborators (2011), the first blood sample (basal CORT) was obtained immediately after removing the bird from the cage (before 3 min); the second blood sample was obtained after a 30-min contention period in a plastic tube (corticosterone level induced by contention) in the same animals. Blood samples were obtained during the first 5 h of the light phase (from 8 to 13 am); because of the time limitation, blood samples were obtained from Day 10 to Day 13. Samples were stored on ice until the end of the sampling period and were then centrifuged for 5 min at 8000 rpm to separate the plasma from red blood cells. The plasma was aspirated with a Hamilton syringe and stored at −80°C until assayed for total CORT content (University of California, Davis). The red blood cells were stored at −80°C for subsequent GSH total assay.

### Plasma corticosterone and GSH levels

CORT level (i.e. concentration) in plasma was determined using direct radioimmunoassay following the method described by Wingfield and Farner (1975) and Wingfield and collaborators (1992). To determine the efficiency of hormone extraction from the plasma, 20 μL of 2000 cpm of tritiated CORT was added to all samples and incubated overnight. Hormones were extracted from the plasma using freshly re-distilled dichloromethane. The aspirated dichloromethane phase was evaporated using a stream of nitrogen at 45°C. Samples were then reconstituted in phosphate-buffered saline with gelatin. All samples were run in duplicate; intra-assay variation was 9.51%, and recovery was 78.49%. Plasma volumes of the samples varied from 2 to 35 μL; we excluded four quail from the traffic noise group and six from the rural noise group because the volume of plasma was too small for CORT determination.

Total GSH concentration was determined in 4-fold diluted erythrocyte lysates using a commercially available kit (Cayman Chemical, Ann Arbor, MI, USA; Catalog no. 703002) according to the manufacturer’s instructions. The results are expressed in μmol·L^−1^ (μM) lysate.

### Data analysis

Data analysis was performed in R 3.3.1 software (R Development Core Team), using linear mixed effect (LME) models fitted by restricted maximum likelihood (nlme package). To meet the assumptions of the LME, the response variables body weight (g) after treatment, TI (s) and GSH (μM) were transformed for normality when needed; all model residuals were normally distributed. Treatment was the fixed factor while cage was a random factor.

To compare if baseline CORT (ng/mL) differed from CORT level at 30 min (stress responsiveness), we used linear mixed models with repeated measures (the individual as a sampling unit). Treatment was the fixed effect while cage was used as a random effect.

Finally, we correlated CORT levels, GSH levels and TI performing a path analysis (Sokal and Rohlf, 1995); we tested the hypothesis that CORT influenced GSH and that GSH influenced TI (i.e. CORT > GSH > TI) separately for the traffic noise and rural noise groups. Data are reported as mean ± SE.

## Ethics note

The study (animal transportation, housing conditions and experimental procedure) was carried out with the approval of the Institutional Committee for the Care and Use of Animals, University of Chile (No. 18112-FCS-UCH).

## Results

### Body weight and TI

There was no significant difference in the final weight of quail at the end of treatment (traffic noise group = 47.76 ± 4.82 g, rural noise group = 48.91 ± 4.79 g) (*t*_12_ = 0.69, *P* = 0.49). There was no significant difference (*t*_12_ = −0.32, *P* = 0.75) in TI behaviour between the traffic (29.95 ± 25.95 s) and rural noise groups (26.06 ± 31.53 s).

### GSH and CORT levels

**Figure 1:**
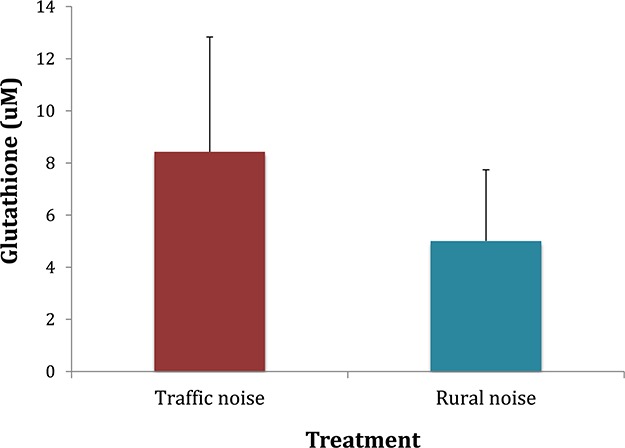
Average glutathione levels (μM) (± SD) in the traffic noise (*N* = 16) and the rural noise (*N* = 14) groups

The traffic noise group presented higher levels (8.43 ± 4.04 μM) of GSH than the rural noise group (5.00 ± 2.73 μM) (*t*_11_ = −2.29, *P* = 0.04) (Fig. 1).

**Figure 2:**
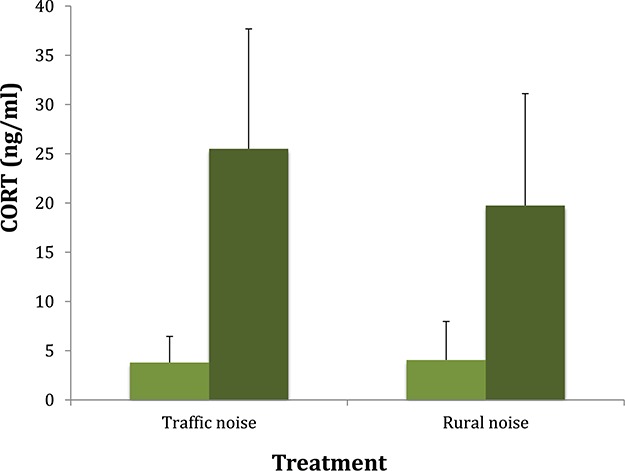
Average baseline (light green) CORT levels (ng/mL) (± SD) and stress response (dark green) in the traffic noise and the rural noise groups

There was a significant difference between group CORT levels both at baseline and at 30 min (stress response) (*F*_1,28_ = 34.65, *P* < 0.001). Although the interaction term between the time when CORT levels were obtained (baseline CORT *vs*. CORT at 30 min) was not significant, levels of CORT at 30 min tended to be higher in the traffic noise group (*F*_1,28_ = 3.30, *P* = 0.08) (Fig. 2).

### Path analysis

**Figure 3:**
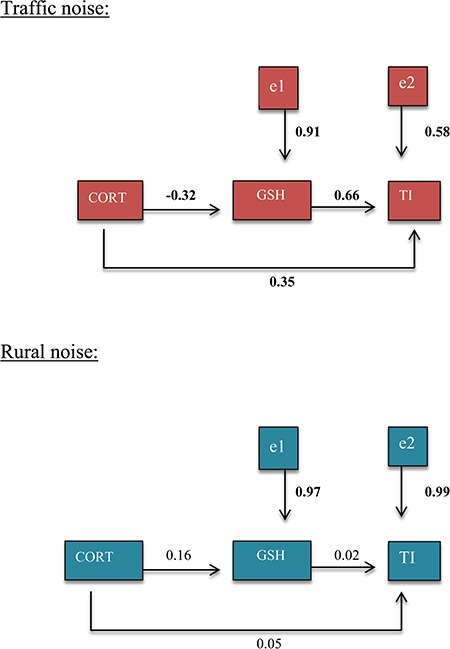
Path analysis of the relationships between the stress response (CORT) level, glutathione (GSH) level and tonic immobility (TI). Also shown are the residuals (e1, e2), which combine all unexplained effects and measurement errors. The path coefficients indicate the strength of the relationships between various pairs of variables when the influences of other variables are accounted for. Values in bold indicate statistically significant path coefficients (*P* < 0.05)

We tested the hypothesis that baseline CORT affects GSH level and how this affects TI. Because we observed a trend toward higher stress response in the traffic noise treatment, we used the stress response in our path analysis. We performed path analysis separately by treatment. For the traffic noise treatment, the stress response had a negative effect on GSH (*pc* = −0.30); GSH had a positive and significant effect on TI (*pc* = 0.66), and the stress response had a positive and significant effect on TI (*pc* = 0.35) (Fig. 3). For the rural noise treatment, the stress response had a positive effect on GSH (*pc* = 0.16), GSH had a positive and weak effect on TI (*pc* = 0.02) and the stress response had positive and weak effect on TI (*pc* = 0.05) (Fig. 3).

## Discussion

### Baseline CORT and stress response

Our first objective was to evaluate the effect of traffic noise on baseline CORT and stress response in quail chicks. We observed similar baseline CORT in the traffic noise and rural noise groups and a trend toward greater stress response in the traffic noise group (*P* = 0.08). In studies related to urban noise, Kleist *et al.* (2018) and Crino *et al.* (2011) reported an increase in the stress response. However, in these studies basal CORT decreased, while in our study basal levels remained the same between the groups and what increased was the level of CORT at 30 min. The study of Crino *et al.* (2013) reported similar baseline CORT levels as in our study, but they reported a decrease in the stress response in the traffic noise group. It is interesting to note that the study of Crino *et al*. (2013) is similar to ours in the sense that they used playback experiments. The other study that performed a playback experiment was that of Angelier *et al.* (2016), who observed no change in CORT level. We found a trend toward higher values at 30 min in the urban traffic noise (*P* = 0.08); the lack of a significant difference could be because (i) the traffic noise was not enough to elicit a significant difference, for example other studies that compared GC values between rural noise and urban noise used a greater difference between rural noise and urban noise (Crino *et al*. 2013: 44-dB rural noise and 59-dB urban noise and Angelier *et al*., 2016: 43-dB rural noise and 61-dB urban noise); (ii) urban noise was not high enough, for example, in the study of Campo and collaborators (2015) rural noise was 65 dB, while urban noise used was 85–90 dB; or (iii) the lack of significant difference could be because of replicates in our study (one rural and one urban noise enclosure). Because statistically significant difference would imply that levels of anxiety are higher in chicks exposed to traffic noise, future studies could both increase the number of replicates and increase the intensity (dB) of the urban noise treatment.

The fact that basal CORT levels were not different between the groups goes against the prediction of the CORT-fitness hypothesis. This hypothesis predicts baseline CORT to be positively associated with the intensity of habitat disturbance (Bonier *et al.*, 2009) and against the development of hypocortisolism, which could be a coping mechanism that saves an organism from experiencing the severe effects of allostatic overload (Fries *et al.*, 2005). Studies that have reported increased stress response in individuals during development have been cases in which individuals were given CORT (in food: Spencer *et al.*, 2009, oral administration: Crino *et al.*, 2014) or in quail through maternal effect (Hayward and Wingfield, 2003). Although a high stress response could decrease parental care and induce nest abandonment (*Ficedula hypoleuca*: Silverin, 1986) and could decrease survival probability of adults (*Ciconia ciconia*: Blas *et al*., 2007; *Melospiza melodia*; MacDougall-Shackleton *et al*., 2013), the characteristics of the species (e.g. long-lived and short-lived species) together with mechanisms such as micro-evolution, phenotypic plasticity or phenotypic flexibility would determine whether such response to environmental changes (e.g. urban noise) is detrimental or beneficial to that species Angelier and Wingfield, 2013).

### Relationships among CORT, GSH and TI

Our second objective was to investigate whether the strength of the association (path correlations) between CORT levels, GSH levels and TI varied in relation to treatment. We observed that the path correlations were stronger and significant in the traffic noise treatment (between 0.30 and 0.60) than in the rural group (between 0.02 and 0.16). In the traffic noise group, and as expected, we observed a negative association between CORT and GSH and a positive correlation between CORT and TI; contrary to our expectations, we observed a positive correlation between GSH and TI.

As has been observed in other species, higher levels of CORT are related to longer TI duration (Jones *et al.*, 1988; Carli *et al.*, 1979; Kalin *et al.*, 1998), so more reactive individuals (stress response) were the fearful individuals that stayed longer in a static position. It is interesting to note that although we found a trend towards greater response to stress in the traffic group and a positive association between CORT and TI, there was no difference in TI between the traffic noise and rural noise groups. This result is contrary to what was found by Campo *et al*. (2005), who observed higher TI duration in the traffic noise group. One reason may be that the decibels we exposed quail to were not high enough to generate such a difference; in the study of Campo *et al.* (2005), the decibel range was between 85 to 90 dB, while in our study it was between 61.9 and 73.4 dB. Therefore, it is possible that an increase in dB could have a greater effect in the response to stress, and since we found a positive association between the levels of CORT and TI (Fig. 3, *cp* = 0.35), we observed longer duration of TI.

Although we found what we expected in terms of the negative association between CORT and GSH (given that CORT decreases GSH), and given that there was a tendency for greater stress response in the noise group, we expected to find lower levels of GSH in the noise group. However, we observed higher levels of GSH in the noise group. One possibility is that the more fearful individuals (positive association between CORT and TI) had an increased heart rate and elevated respiratory rate, which in turn increased free radicals (Costantini *et al.*, 2011), thus generating an upregulation of the antioxidant system.

To our knowledge, the only study that has evaluated levels of GSH comparing urban and rural sites is that of Isaksson (2005), where she noted that the ratio between oxidized and reduced glutathione (GSSG:GSH) in urban individuals of great tits (*Parus major*) was higher than in rural counterparts, suggesting elevated stress in the urban environment. However, the total GSH was similar between the urban and rural environment. Total GSH indeed reflects a general mobilization of the GSH system in response to oxidative stress and may indicate long-term upregulation of the GSH reservoir, so that higher total GSH levels indicate higher oxidative stress. Apparently, this upregulation can be as fast as 5 days (Stephensen *et al.*, 2002), in our study 8 days.

Finally, why the path correlations were stronger in the traffic noise group than in the rural noise group should be investigated in future studies. What these strong associations may reflect is higher CORT and GSH values. If this is the case, we should have observed greater responses and fitness consequences in urban environments.

We conclude that the acoustic environment perceived during development has implications for physiology and behaviour, and as more research is done on this topic, the need for sustainable urban planning will be clearer.

## Funding

This study was supported by ‘Dirección General de Investigación’ (project: DI-12-17/RG), Universidad Andres Bello.

## Acknowledgements

V.Q. conceived and designed the study. V.Q., R.F. and M.P. carried out the study. J.C.W. and V.Q. performed the hormone assays. All authors reviewed and revised the article critically and approved the final version.
